# Before respiratory muscle weakness is attributed to a stroke, alternative causes must be considered and thoroughly ruled out

**DOI:** 10.1590/1806-9282.20241422

**Published:** 2025-05-02

**Authors:** Josef Finsterer

**Affiliations:** 1 Neurology and Neurophysiology Center – Vienna, Austria.

Dear Editor,

We were interested to read the article by Yildiz et al. on a prospective study of respiratory muscle strength in stroke patients, in which maximal inspiratory pressure (MIP) and maximal expiratory pressure (MEP) were measured in 171 patients with chronic stroke compared to 32 healthy controls matched for age and gender^1^. Stroke patients were found to have lower MIP and MEP than healthy controls^1^. It was concluded that patients with chronic stroke can suffer from significant respiratory muscle weakness, which requires adaptation of rehabilitation programs for stroke patients^1^. The study is impressive, but some points should be discussed.

The first point is that the spectrum of the causes of respiratory muscle weakness is broad and that these various causes must be ruled out before respiratory muscle weakness is attributed to chronic stroke. The causes of respiratory muscle weakness to be ruled out include electrolyte disturbances, neuromuscular disorders, transmission diseases, polyradiculitis, neuropathy (including critically ill neuropathy), psychiatric illness (depression, delirium), pain, and acidosis or alkalosis.

The second point is that the degree of respiratory muscle weakness after a stroke can depend strongly on the localization of the stroke. The weakness may be different in a stroke in the right or left middle cerebral artery area compared to the basilar artery. If the respiratory center in the brainstem is affected by the stroke, the respiratory drive may be severely impaired. Respiratory muscle strength may also depend on whether the patient has had previous lung or bronchial disease, asthma, pulmonary embolism, allergy, sleep apnea syndrome, or heart disease, including left or right heart failure.

The third point is that the latency between the onset of the stroke and the date of the respiratory muscle examination was only given as a mean value without a range or standard deviation. We should know the minimum and maximum latencies. Since a mean latency of 388 days, as shown in Table 1, is quite long, it cannot be excluded that causes other than the stroke developed in the post-stroke period and were actually responsible for the decreased MIP or MEP. Therefore, we should know how many of the included patients had an intermediate disease after the stroke that could explain the reduction in MIP or MEP.

The fourth point is that the methods state that the stroke cohort and the healthy control group were matched by age and gender. As the two groups were of different sizes (171 vs. 32), it should be explained how the matching was performed.

The fifth point is that despite the indication that the two groups were matched for gender, there was a significant difference in the ratio of male to female in the patient and control groups^1^. In the stroke cohort, the proportion of males was 39%, while in the control group, 56% of the participants were male. This discrepancy should be corrected.

The sixth point is that the recruitment period also included the pandemic^1^. Since severe acute respiratory syndrome-coronavirus-2 (SARS-CoV-2) infection can strongly influence MIP and MEP^2^, we should know how many of the included patients had SARS-CoV-2 infection, its severity, and whether any of them needed mechanical ventilation.

The seventh point is that it was not explained what is meant by chronic stroke. Do the authors mean a history of stroke or do they mean an incomplete stroke? Were only patients with ischemic stroke or also patients with cerebral hemorrhage included in the study?

In summary, it can be said that the index study has limitations that relativize the results and their interpretation. Addressing these limitations could strengthen the conclusions and support the results of the study. Before respiratory muscle weakness in stroke patients after 1 year is attributed to the vascular event, alternative causes must be considered and thoroughly ruled out.
